# An association between poor oral health, oral microbiota, and pain identified in New Zealand women with central sensitisation disorders: a prospective clinical study

**DOI:** 10.3389/fpain.2025.1577193

**Published:** 2025-04-09

**Authors:** Sharon Erdrich, Ingrid C. Gelissen, Momchilo Vuyisich, Ryan Toma, Joanna E. Harnett

**Affiliations:** ^1^ Faculty of Medicine and Health, Sydney Pharmacy School, The University of Sydney, Sydney, NSW, Australia; ^2^ Viome Life Sciences, Bothell, WA, United States

**Keywords:** pain, central sensitisation, migraine, functional abdominal pain, oral health, microbiome

## Abstract

**Introduction:**

The portal to the gastrointestinal tract is the oral cavity, with transient and permanent microbial residents. Oral pathogens are implicated in the aetiology of several chronic conditions. To date, the role of oral health and the oral microbiota in the aetiology of pain in sensitisation disorders have not been explored. Here, we examined associations between self-reported oral health, the oral microbiome, and various pain presentations in women.

**Methods:**

Oral health in women was assessed using the WHO oral health questionnaire. Body pain, migraine, and abdominal pain were determined using validated instruments. Saliva samples were evaluated using metatranscriptomics for relative gene abundance. Demographic and clinical characteristics data were evaluated for relationships between oral health scores, pain measures, and the oral microbiota at three taxa levels.

**Results:**

Participants in the lowest quintiles for oral health were more likely to suffer migraine headaches (*χ*^2^ = 23.24, df 4, *p* < 0.001) and higher body pain scores. Four oral pathogenic species were significantly associated with SF36 bodily pain (*q* < 0.05) after controlling for confounders. Relative abundance of *Gardnerella* (genus) correlated moderately with oral health scores (*ρ* = −0.346, *q* = 0.001), while *Lancefieldella* (genus) and *Mycoplasma salivarius* were associated with migraine.

**Discussion:**

Low oral health scores correlated with higher pain scores. Both were associated with higher relative abundance of oral pathobionts. This suggests a potential role for the oral microbiota in the aetiology of pain experienced by women with migraine headache and abdominal and body pain. These findings prompt consideration of an oral microbiome–nervous system axis.

**Trial registration:**

The study was registered with the Australia and New Zealand Clinical Trials Registry (ANZCTR), registration number ACTRN12620001337965, on 11/12/2020 https://www.anzctr.org.au/, and with the World Health Organisation, UTN: U1111–1258-5108.

## Introduction

Migraine, fibromyalgia, chronic regional pain syndrome (CRPS), irritable bowel syndrome (IBS), and temporomandibular joint disorder (TMJ) are examples of central sensitisation disorders, largely of unknown aetiology. Diagnosis is primarily based on subjective measures and clinical management focuses on symptom mitigation (1).

Pain is subjective, classified according to the site, cause, affected anatomical system (e.g., neuropathic), and chronicity, broadly defined as ‘an unpleasant sensory and emotional experience associated with, or resembling that associated with, actual or potential tissue damage’ (2). Three subcategories further define pain: nociceptive, the most common, resulting from physiological tissue injury and observable activity in the nervous system (via imaging studies); neuropathic (from damaged neural tissue); and nociplastic, occurring in the absence of tissue damage and hypothesised to be associated with nervous system dysregulation (1, 3). Nociplastic pain is implicated in central sensitisation disorders (4).

The chronic central sensitisation disorder fibromyalgia is commonly associated with disturbed digestive function (5) and gastrointestinal microbiome alterations (6). Commonly comorbid, migraine is also associated with nociplastic pain and gastrointestinal dysfunction (7). Flares and mitigation of one of these conditions are predictive of a synchronous effect on the other (8). Fibromyalgia is also commonly comorbid with other hyperalgesic conditions, including TMJ, IBS, bladder, and pelvic pain, thus presenting as a useful model for the study of chronic, idiopathic pain.

People living with chronic pain report lower quality of life, poor sleep, increased stress and anxiety, loss of earning potential, and limitations to physical activity, increasing the risk of other chronic conditions. Chronic idiopathic pain poses further challenges, including experiencing disbelief and stigmatisation, polypharmacy, and risk of substance abuse (9). These challenges predispose to depression and social isolation and hinder access to support and care (1).

The gut–brain axis has been implicated in the pathophysiology of central sensitisation disorders. To date, the role of the mouth, portal to the gastrointestinal tract and harbouring an estimated 600 microbial species (10), is underexplored in pain aetiology. Oral microbes and dietary components translocate to the distal gastrointestinal tract, with microbial communities in one niche predictive of the other (11).

The relationship between periodontal problems and cardiovascular disease has been well examined (12), and attention has recently extended to a wide array of potential progenitors, including microbes associated with root canal-filled teeth (13). While the American Gut Project demonstrated a role for oral microbes in migraine (14), links between OH and the oral microbiota with migraine and other pain disorders more prevalent in women have not yet been explored.

The objective of this study was to explore associations between self-reported OH, the oral microbiome, and migraine, functional abdominal pain, and body pain in women.

## Materials and methods

A secondary analysis of data obtained during a prospective observational study involving non-smoking women without diabetes or chronic inflammatory disorders, who were recruited from the community in Auckland, New Zealand, from December 2021 to December 2022.

**Figure 1:**
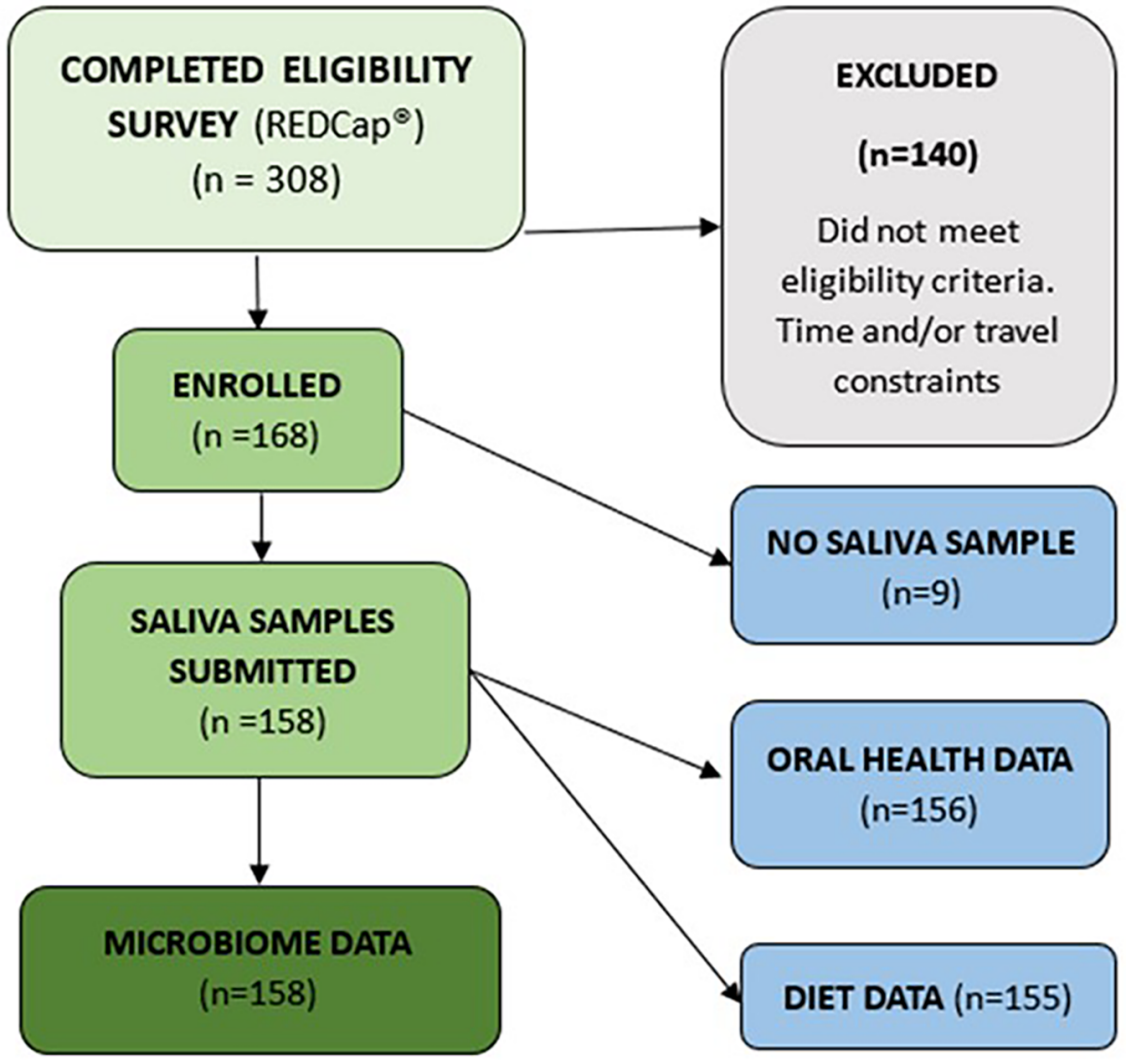
Recruitment flow diagram.

One hundred and sixty-eight women were enrolled in the study. The study protocol details the primary study from which these data were obtained, outlining inclusion and exclusion criteria and testing procedures, which are published in BMC Musculoskeletal Disorders (15). Here, we provide additional detail of the measures used in this secondary analysis. From those enrolled, oral microbiome data were available for 158 and oral health data for 156 (see Figure 1). The data from *n* = 158 are included in the analysis.

For this analysis, data measuring body pain, migraine, abdominal pain, oral health (OH), and the oral microbiome were evaluated. For body pain, the short-form 36 (SF36) domain ‘bodily pain’ (SF36-BP), a self-reported measure of pain intensity and associated disability over the previous 4-week period, with scores inverted. Thus, an original score of 20 (indicating more pain) became 80 on the revised scale, so a high score was consistent with more pain. The widespread pain index (WPI), from the ACR 2016 fibromyalgia diagnostic criteria (16), was also employed, as women with this central sensitisation disorder were included in the analysis. The WPI is a tally of specific body sites and regions in which pain was experienced in the previous week (16). The International Headache Society criteria were applied to the headache symptom questionnaire (HSQ) data using a scoring algorithm, identifying current migraine and sub-typing for frequency as detailed and validated by van der Meer et al. (17). The functional bowel disorder severity index (FBDSI), which measures abdominal pain intensity on a visual analogue scale, constancy of the pain, and the number of associated visits to healthcare providers, was used. The FBDSI is endorsed for use in painful functional bowel conditions such as functional abdominal pain syndrome (18).

Oral health was evaluated using the World Health Organization (WHO) OH questionnaire for adults (OHQ) (19). This tool embraces the multidimensional nature of OH, without assigning items to domains. Thus, a composite score was created as follows: questions were assigned to one of seven categories: oral care (5 items), OH affects oral function (4 items), OH affects psychosocial well-being (8 items), diet and lifestyle influences on OH (20 items), OH history (10 items), OH problems (12 items), and self-awareness of OH problems [2 items, from Locker's validated global survey (20)]. Items were scored on Likert scales (ranging from 3 to 6 points, depending on the question) and tallied, such that a high score (maximum 308 points) was predictive of good OH. Details are provided in Supplementary Table S11.

Due to the known association between diet and OH, diet was evaluated using the photometric tool, Diet ID, from which estimates of total dietary sugars and added dietary sugar intake (grams/day) were obtained.

Study participants provided saliva samples, ensuring at least 4 weeks since the use of any antibiotics and 2 weeks since probiotic use, and were consuming their usual diet. Saliva samples were collected upon arising and before eating and teeth-cleaning, as per the published protocol (15). Samples were stored at −20°C and then transferred to a −80°C freezer until analysis, using meta transcriptomic technology at (Viome Life Sciences, Seattle, WA, United States).

Participant demographic and clinical characteristic data were evaluated for relationships between OH scores, the SF36-BP, WPI, migraine, and FBDSI scores before examining these data against the oral microbiome.

### Sample size calculation

A sample power calculation was conducted using G*Power v3.1.9.7 (21) to establish that the study (*n* = 156) was adequately powered to detect primary correlation (*r* = 0.230, 82.8% power), chi-square (*χ*^2^ = 11.07, 83.9% power), and linear effects with three predictors (effect size *f*^2^ = 0.15, 95% power) at *α* = 0.05.

### Microbiome data processing

After filtering out low prevalence features (i.e., detected in ≤10% of the total cohort) (22), the proportional abundance of RNA within phyla, genus, and species per sample was converted to a matrix of read counts using the total number of microbial reads per sample. Taxa with a total of 50 reads or less across all individuals were filtered out.

For principal components analysis (PCA), hierarchical clustering was used: counts of 0 were replaced by an imputed estimate using the *z*Compositions package (23). The centred log ratio (CLR) was calculated for each read count using the geometric mean for centring. PCA plots and hierarchical clustering outputs were examined for evidence of batch effects.

For differential abundance and generalised linear models (GLMs), the following read count matrix was used: phyla, genus, or species with a mean read count of less than 1 across all samples were filtered out. Differential expression and GLMs were performed using ALDEx2 (24) using 128 Monte Carlo instances generated from the CLR transformed read count matrix.

Within the cohort, GLMs were performed to examine the association between the differential abundance of the microbiota at each taxa level and the clinical variables of interest. After adjustment for age, BMI, and added dietary sugar, all with known influences on OH (25). As dietary intake of sugar-containing food contributes (negatively) to the composite OH score, GLMs examining the effect of OH on the microbiome excluded added dietary sugar intake to avoid potential issues due to multicollinearity.

Missing data were handled case-wise in the analysis. For binary clinical variables of interest, expected *p*-values for Welch's *t*-test/Wilcoxon rank sum and the standardised effect size were used to examine the evidence for the association of the clinical variable of interest with the outcome after adjustment for covariates. For continuous clinical variables of interest, the expected *p*-value for the *t*-test that the coefficient 0 was used.

The Shannon diversity index was calculated from read count matrices using vegan (26). Correlations were calculated between continuous clinical variables of interest and each alpha diversity metric. For binary variables of interest, a paired *t*-test was performed to compare alpha diversity metrics with the cohort variably divided for the main clinical features.

Between-group differences in microbial abundance were evaluated using Pearson's chi-square (or Fisher's exact test) for categorical variables and either the *t*-test for mean values or the Mann–Whitney *U* (M­–W *U*) test was used for statistical analysis of continuous variables, depending on the normality of the data. For comparisons between groups, both means and medians are shown due to the low read numbers of some microbes. Comparisons within a group were conducted using the Kruskal–Wallis *H* test with Bonferroni correction, after dividing participants into quintiles, or grouped according to the tool used. Correlations were assessed using Spearman's rho and confidence intervals set at 95% by Caruso and Cliff's method. A two-tailed *p*-value of <0.05 was considered statistically significant. The Benjamini–Hochberg (BH) procedure for correction of false discovery rate (FDR) for multiple comparisons was employed, with an adjusted *α* of 0.1. All data processing and statistical analyses were performed with Microsoft Excel, IBM Statistical Package for Social Science (SPSS) version 28 software, and R version 4.2.1.

## Results

The baseline characteristics of the cohort (*n* = 158) included in the analysis, such as oral health and pain measures, are detailed in Table 1.

**Table 1:** Characteristics of the cohort (*n* = 158).

Characteristic	Mean (SD)	Median^a^ (IQR)	*N* (%)
Age (range, 19–74)	45.7 (13.0)		
BMI kg/m^2^	28.5 (7.5)	26.7 (10)	
- Diet quality (HEI)	81.0 (19.3)	88.0 (21)	
- Total dietary sugars (g/day)	78.2 (24.3)	75.6 (27)	
Added dietary sugar (g/day)	20.6 (28.9)	5.1 (25)	
Ethnicity: primary group [secondary group]			
- New *Z*ealander			112 (71.8)
- European			33 (21.2)
- Māori			1 (0.6), [8 (16.0)]
- Asian			3 (1.9) [3 (1.9)]
- Other or undisclosed			7 (4.5)
Oral health (*n* = 156)			
Oral health score	193.8 (16.5)	196.0 (24)	
- Domain 1: oral health history and state of teeth	19.3 (4.3)	20.0 (8)	
- Domain 2: oral health problems	36.0 (8.4)	37.5 (13)	
- Domain 3: oral care (preventative behaviour)	16.0 (2.7)	16.0 (4)	
- Domain 4: oral health affects oral function	10.2 (2.3)	11.0 (3)	
- Domain 5: oral health affects psychosocial well-being	23.0 (2.1)	24.0 (1)	
- Domain 6: diet and lifestyle influences	83.3 (5.5)	83.0 (7)	
- Domain 7. self-awareness of oral problems	6.0 (2.1)	6.0 (4)	
Pain measures			
Widespread pain index	8.8 (6.2)	10.0 (12.0)	
SF36 bodily pain (inverted)	43.2 (27.6)	50.0 (58)	
Functional bowel disorder severity	31.2 (51.6)	9.0 (33)	
Migraine symptom scores	4.5 (2.9)	5.0 (5.0)	
Meets fibromyalgia criteria			106 (67.1)
Meets migraine criteria			76 (48.7)
- Frequent migraine			48 (63.2)
- Chronic migraine			14 (18.4)
- Infrequent migraine			14 (18.4)

HEI, healthy eating index.

^a^ Medians presented for results of non-parametric data only.

### Oral health, diet, and BMI

Of the included 158 participants, 156 provided oral health data. The correlation between Locker's validated two-question tool (Domain 7 of the OHQ) and the overall oral health score with these two questions removed was *ρ* = 0.553 [*p* < 0.001, 95% CI (0.43–0.66)] indicating moderate congruence. Subsequent analysis was conducted using the full OH survey, which includes Locker's two questions. Self-reported halitosis (an oral health problem item in the OH questionnaire) was predictive of a low OH score, with higher scores in those for whom halitosis had either never been a problem or was a past problem, now resolved for at least 12 months [*H* = 41 (4), *p* < 0.001].

The HEI, a measure of diet quality, ranged from 18 to 98 out of a maximum score of 100 over the 155 participants for whom these data were available. The HEI was weakly correlated with oral health *ρ* = 0.205 [*p* = 0.01, 95% CI (0.05–0.35)], and inversely with BMI [*p* = 0.03, 95% CI (−0.32– −0.02)]. No association was seen with age. The OHQ includes items that specifically assess the intake of added dietary sugar (refer to Supplementary Table S1).

### Oral health and pain

Self-reported overall OH scores were significantly (*p* < 0.001) and inversely correlated with each of the measures of pain (Supplementary Table S2).

### Oral health and body pain

**Figure 2:**
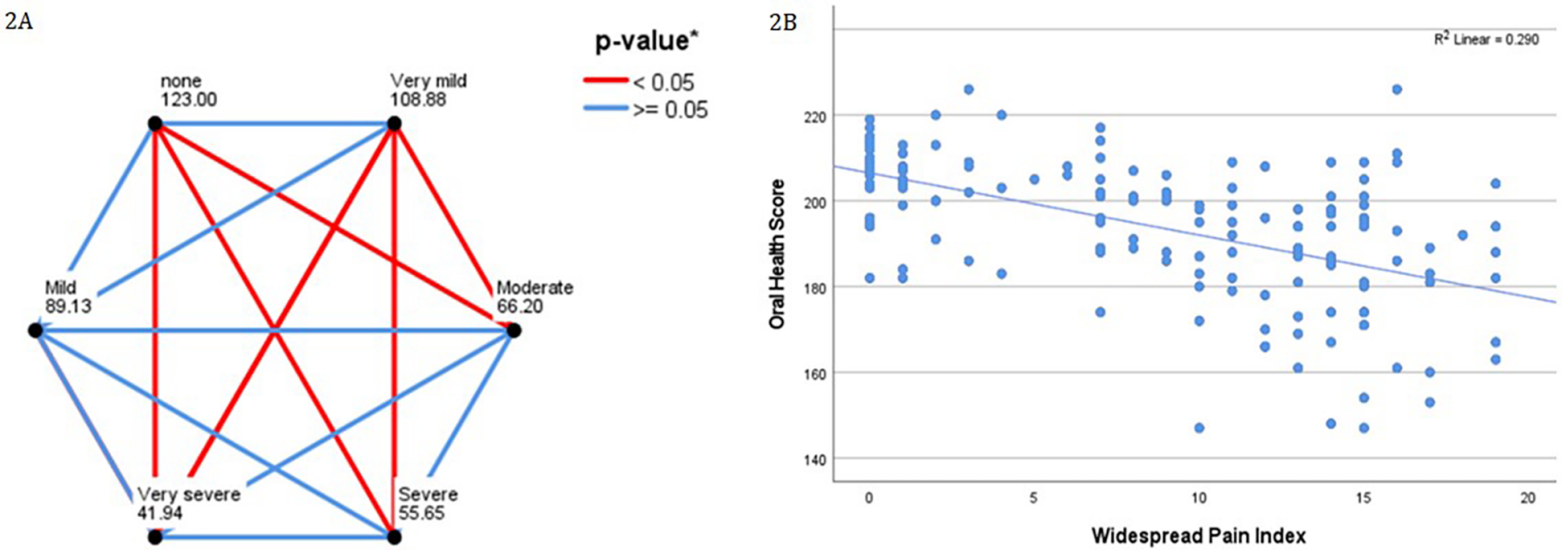
**(A)** Pairwise comparisons of oral health scores with SF-36 bodily pain score, when grouped by quintile. Participants scoring zero for the SF36-BP are compared separately. Each node shws the average rank for SF36 bodily pain by quintile. *p-values after Bonferroni correction for multiple comparisons. **(B)** Demonstrates increasing scores on the widespread pain index (range, 0–19) correlating with lower oral health scores (range, 147–226).

The SF36 was completed by 156 participants. Seventeen (10.8%) scored 0 for bodily pain (of a possible 100) on the SF36-BP. When the remaining participants were divided into quintiles according to the bodily pain score, 28 (17.7%) were in the very mild category, 16 (10.1%) mild, 49 (31.0%) moderate, 37 (23.4%) severe, and 9 (5.7%) very severe. Pairwise comparisons were conducted and are presented in Figure 2A. The correlation between the WPI and OH scores is shown in Figure 2B.

### Oral health and migraine

**Figure 3:**
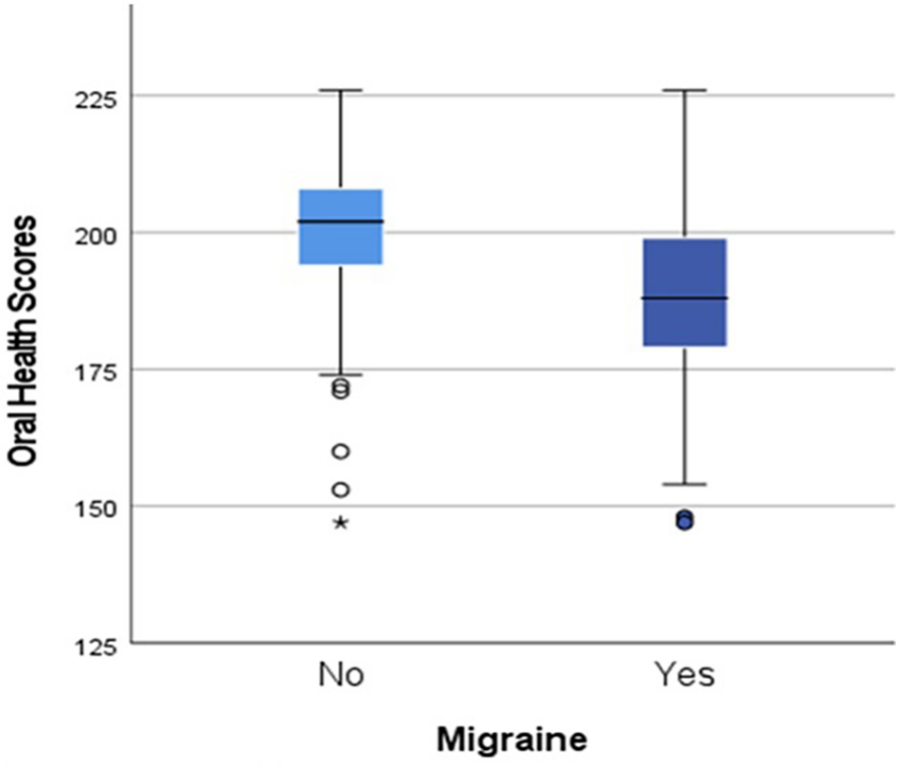
Differences in oral health scores by migraine status, *p* < 0.001.

The total OH scores of women with migraine were significantly lower than those without migraine [mean (SD) 188 (16.8), cf. 199 (14.7) *p* < 0.001, 95% CI (−15.7, −1.6)] as shown in Figure 3.

**Figure 4:**
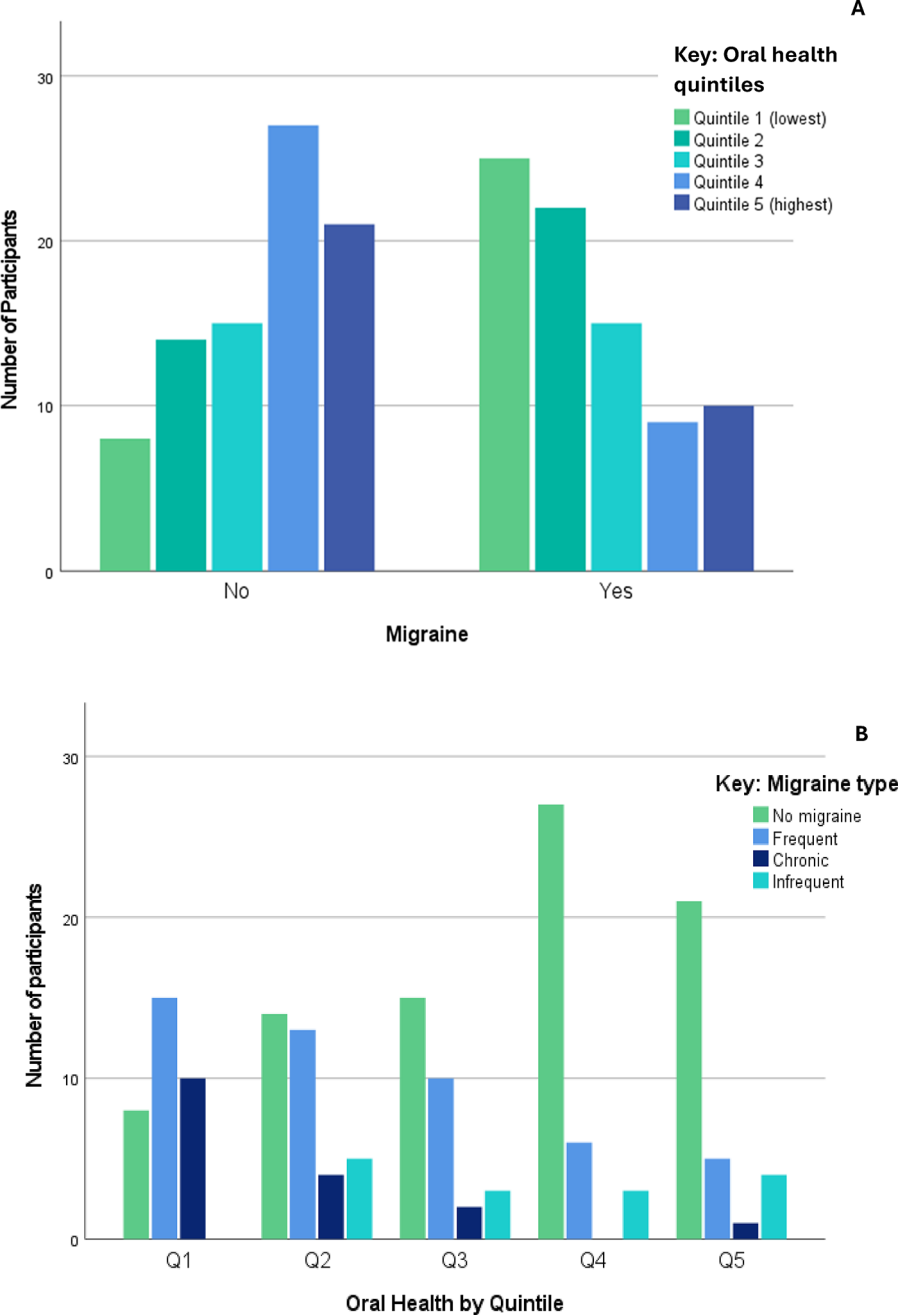
Migraine headache **(A)** and migraine subtypes **(B)** by oral health quintiles.

Participants in the lowest OH quintiles were more likely to suffer migraine headaches [*χ^2^* = 23.24 (4), *p* < 0.001], as shown in Figure 4A. When grouped by quintile according to OH scores, 58% of those with migraine and 25% without migraine were in the lowest two OH quintiles, while 21% of those with migraine and 54% without migraine were in the highest two quintiles.

Figure 4B presents the associations between OH and migraine subtypes—lower OH groups were more commonly affected by ‘frequent’ and ‘chronic’ migraine [*χ*^2^ 34.8 (12), *p* < 0.001]. A Kruskal–Wallis *H* test showed that overall, the association between migraine type and OH was significant [*H* = 24.5 (3), *p* < 0.001], with the most significant differences seen between those without migraine and the frequent and chronic groups (all *p* ≤ 0.001 after Bonferroni correction).

Regression analysis indicated that OH was a statistically significant predictor of frequent and chronic migraine. Higher OH scores were associated with a reduced likelihood of having frequent (OR = 0.95) or chronic (OR = 0.92) migraine (both *p* < 0.001). Higher BMI significantly increased the odds of frequent migraine (OR = 1.10, *p* = 0.003) and chronic migraine (OR = 1.13, *p* = 0.004). No effect was seen for age.

### Oral health and abdominal pain

**Figure 5:**
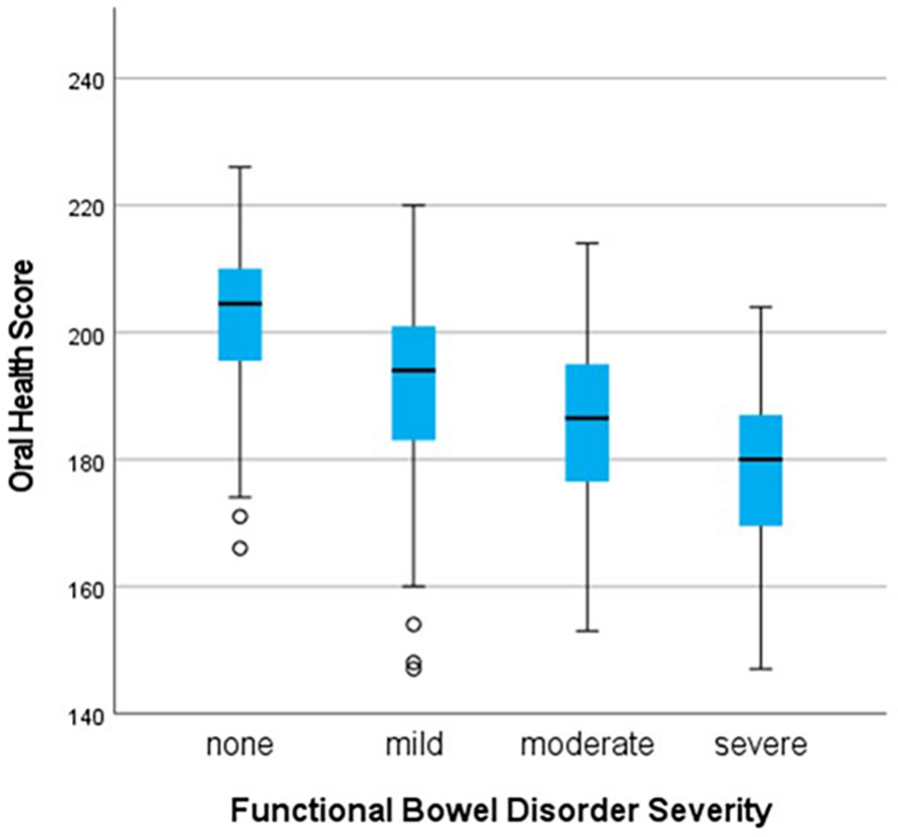
Comparison of oral health scores by severity groupings for functional bowel disorders, demonstrating that as oral health scores decrease, severity of functional bowel disorder-related pain increases. *All *p*-values <0.001 after Bonferroni correction for multiple comparisons.

A significant difference in OH scores was seen according to the severity of FBDSI [*H* = 42.5 (3), *p* < 0.001]. Pairwise comparisons (Kruskal–Wallis *H* test) were significant between the group without any functional bowel disorder (*n* = 64), and each of the mild (*n* = 54), moderate (*n* = 20), and severe (*n* = 15) groups after Bonferroni correction as shown in Figure 5.

### Oral health, pain, and the oral microbiome

A total of 31 phyla were detected in the saliva samples across all participants. These were 13 bacteria, six viruses, eight eukaryotes (including two fungi and four protists), two archaea, and two metagenomic assemblies (MAGs). There were 657 genera and 1,747 species. After filtering out taxa and microbes with low prevalence and low reads and non-informative MAGs, 14 phyla (10 bacteria, two fungi, one archaeon, and one virus), 120 genera and 289 species remained for the analysis.

### Microbial species and oral health

The relative abundance of 52 oral species was significantly associated with OH scores, meeting the FDR *α* of ≤0.10. Thirty of these were significant at *q* < 0.05. Inverse correlations were seen for 16 species, with the strongest correlation coefficient seen for *Gardnerella vaginalis* (*ρ* = −0.379). Positive correlations were seen for multiple species associated with a normal oral microbiome (Supplementary Table S3).

Applying the linear model (adjusting for age and BMI), 20 species were significantly associated with OH after FDR adjustment (Supplementary Table S4).

Age was positively associated with relative abundance for *Haemophilus parahaemolyticus*, *H. sputorum*, and *Stomatobaculum longum* (all *p* < 0.05 before FDR), but not after adjustment (*q* > 0.20).

### Microbial species and body pain

After adjusting for age, BMI, and added dietary sugar, 32 species were associated with body pain, as measured by the SF36. Of these, fourteen were correlated with the SF36-BP and are annotated accordingly in Table 2.

**Table 2:** Bodily pain (SF36) associated with relative abundance of oral species, after adjusting for age, BMI, and added dietary sugar.

Oral species	Estimate	SE	*p*-value	*q*
*Actinomyces* sp. oral taxon 171	−0.022	0.01	0.005	0.28^b^
*Aggregatibacter segnis*	−0.039	0.01	<0.001	0.14^b^
*Aggregatibacter* sp. 2125159857	−0.025	0.01	0.03	0.60
*Anaeroglobus geminatus*	0.026	0.01	0.03	0.42^a^
*Capnocytophaga sputigena*	−0.016	0.01	0.03	0.58
*Dialister pneumosintes*	0.036	0.00	<0.001	0.04^a^
*Fusobacterium massiliense*	−0.03	0.01	0.04	0.56
*Fusobacterium nucleatum*	0.016	0.00	<0.001	0.03^a^
*Haemophilus parahaemolyticus*	−0.039	0.02	0.01	0.42^b^
*Haemophilus paraphrohaemolyticus*	−0.038	0.01	0.01	0.29^b^
*Lancefieldella parvula*	0.015	0.01	0.03	0.43
*Neisseria cinerea*	−0.026	0.01	0.04	0.61
*Neisseria lactamica*	−0.03	0.01	0.02	0.43^b^
*Neisseria mucosa*	−0.03	0.01	0.01	0.34^b^
*Neisseria polysaccharea*	−0.029	0.01	0.02	0.40^b^
*Neisseria subflava*	−0.03	0.01	0.02	0.44^b^
*Parvimonas micra*	0.022	0.01	<0.001	0.05^a^
*Prevotella denticola*	0.017	0.01	0.003	0.15^a^
*Prevotella enoeca*	0.013	0.01	0.04	0.52^a^
*Prevotella fusca*	0.017	0.01	0.03	0.52
*Prevotella intermedia*	0.013	0.01	0.01	0.34
*Prevotella oralis*	0.038	0.01	0.01	0.26^a^
*Prevotella oris*	0.013	0.01	0.01	0.32^a^
*Pseudoleptotrichia goodfellowii*	−0.036	0.01	0.02	0.41^b^
*Schaalia meyeri*	0.023	0.01	0.04	0.48^a^
*Selenomonas* sp. oral taxon 920	0.015	0.01	0.03	0.64
*Solobacterium moorei*	0.018	0.00	<0.001	0.02^a^
*Streptococcus gordonii*	0.016	0.01	0.01	0.34
*Streptococcus parasanguinis*	0.011	0.01	0.04	0.53
*Streptococcus* sp. LPB0220	0.014	0.01	0.02	0.35
*Veillonella dispar*	0.015	0.01	0.02	0.35

^a^ Positively correlated with SF36-BP scores.

^b^ Inversely correlated with SF36-BP scores, *q* = adjusted *p*-value after Benjamini–Hochberg correction.

A significant positive association was seen between age and *Selenomonas* sp. oral taxon 920 (est. 0.04, *SE* 0.01, *t* *=* 2.80, *p* = 0.01), which was insignificant after FDR.

SF36-BP scores were correlated with 28 species (correlation coefficient less than −0.200 or >0.200). Of the 25 that remained significant (*p* ≤ 0.10) after FDR, 13 were positively, and 12 were inversely correlated, as shown in Supplementary Table S5.

As the four species with the strongest inverse correlations (*Parvimonas micra*, *Solobacterium moorei*, *Dialister pneumosintes*, and *Prevotella denticola*) retained significance after FDR in the GLM are all known to be associated with oral problems and extraoral infections, an additional correlational analysis was performed to assess the relationships between these species. All correlation coefficients were *ρ* > 0.350 except between *Parvimonas micra* and *Prevotella denticola* (*ρ* = 0.263). All had *p* < 0.001 after applying the Benjamini–Hochberg adjustment for multiple comparisons.

### Microbial species and migraine

In the GLM, *M. salivarium* met initial *α* (est. 1.47, SE 0.63, *t* = 2.3, *p* = 0.03), and a negative effect of BMI was seen (est. −0.01, SE 0.03, *t* = −0.5, *p* = 0.03). Both were insignificant after FDR. Other associations with migraine were observed for *Aggregatibacter* sp. 2125159857 (est. −1.57, SE 0.60, *t* = −2.6, *p* = 0.012), *Capnocytophaga* sp. FDAARGOS_737 (est. −1.15, SE 0.50, *t* = −2.3, *p* = 0.02), *C. leadbetteri* (est. −1.19, SE 0.50, *t* = −2.4, *p* = 0.02), *Fusobacterium nucleatum* (est. 0.51, SE 0.23, *t* = −2.2, *p* = 0.03), *Prevotella intermedia* (est. 0.57, SE 0.28, *t* = −2.0, *p* = 0.049), and *Veillonella parvula* (est. 0.71, SE 0.29, *t* = −2.5, *p* = 0.01), none of which were significant after FDR.

Relative abundance of *Mycoplasma salivarium* (phyla: *Mycoplasmatota*) was significantly higher in migraineurs than those without migraine (M 0.78, SD 2.1, Md 1.0, IQR 2.7 cf. M −0.36, SD 2.3, Md 0.26, IQR 2.5, *Z* = −3.30, *p* < 0.001). A Kruskal–Wallis *H* test indicated an overall significant association with migraine type [*H* = 16.2 (3), *p* = 0.001], with frequent vs. no migraine significant after Bonferroni correction (*H* = −33.0, SE 18.5, *p* < 0.001).

Of the nine species with correlation coefficients of <0.200 or >0.200, only *Mycoplasma salivarium* remained significantly correlated with migraine scores after applying the FDR correction [*ρ* = 0.332, *p* < 0.001, 95% CI (0.18, 0.47), *q* = 0.01].

For migraine type, *Lancefieldella parvula*, *L. rimae*, *Prevotella salivae*, *Streptococcus parasanguinis*, *Streptococcus* sp. LPB0220, *Streptococcus* sp. NPS 308, *Veillonella atypica*, *V. dispar*, and *V. nakazawae* were associated with all three migraine types (all *p* < 0.04). None were significant after FDR, with the strongest relationship seen for *V. atypica* and chronic migraine (est. 8.2, SE 2.5, *t* = 3.2, *q* = 0.19). No associations were seen for the effect of age, BMI, or added dietary sugar.

### Microbial species and abdominal pain

Applying the regression model, the FBDSI score was associated with 10 species: *Fusobacterium hwasookii* (est. 6.8, SE 2.8, *t* = 2.4, *p* = 0.02), *Leptotrichia* sp. oral taxon 212 (est. 7.8, SE 3.2, *t* = 2.4, *p* = 0.02), *Streptococcus equi* (est. 6.9, SE 2.4, *t* = 2.9, *p* = 0.005), *S. gallolyticus* (est. 7.8, SE 3.8, *t* = 2.0, *p* = 0.04), and *Veillonella atypica* (est. 4.5, SE 2.8, *t* = 2.0, *p* = 0.02). None were significant after adjustment for FDR.

Functional bowel disorder severity scores were weakly correlated with relative abundance of 10 species, with correlation coefficients between 0.200 and 0.250. These were *Fusobacterium hwasookii*, *F. nucleatum*, *Leptotrichia* sp. oral taxon 212, *Solobacterium moorei*, *Streptococcus equi*, *S. gallolyticus*, *Streptococcus* sp. *LPB0220*, *Veillonella atypica*, and *V. dispar*. All met initial *α*, with *p* < 0.01, and none were significant after FDR.

### Microbial genera and oral health

After filtering the 282 low reads and low abundance genera (which constituted 0.05% of the total), 120 genera remained. Of these, 10 comprised 77% of the total makeup of the oral genus pool (Supplementary Figure S1).

Applying the GLM, OH was associated with the following genera: *Alloprevotella* (est. 0.01, SE 3.6, *t* = 3.6, *p* = 0.001, *q* = 0.04), *Gardnerella* (est. 0.02, SE −3.7, *t* = −4.0, *p* = 0.001, *q* = 0.05), *Granulicatella* (est. 0.02, SE −4.0, *t* = −3.2, *p* = 0.002, *q* = 0.06), *Lactobacillus* (est. −0.07, SE −3.7, *t* = 0.02, *p* = 0.001, *q* = 0.03), *Porphyromonas* (est. 0.03, SE 0.01, *t* = 3.1, *p* = 0.002, *q* = 0.09), and *Simonsiella* (est. 0.11, SE 3.5, *t* = 3.5, *p* = 0.001, *q* = 0.05). The OH score was also positively associated with *Neisseria* (est. 0.03, *SE* 0.01, *t* = 2.9, *p* = 0.006, *q* = 0.12), which was insignificant after FDR adjustment. Age (but not BMI) had a positive, marginally significant effect.

**Figure 6:**
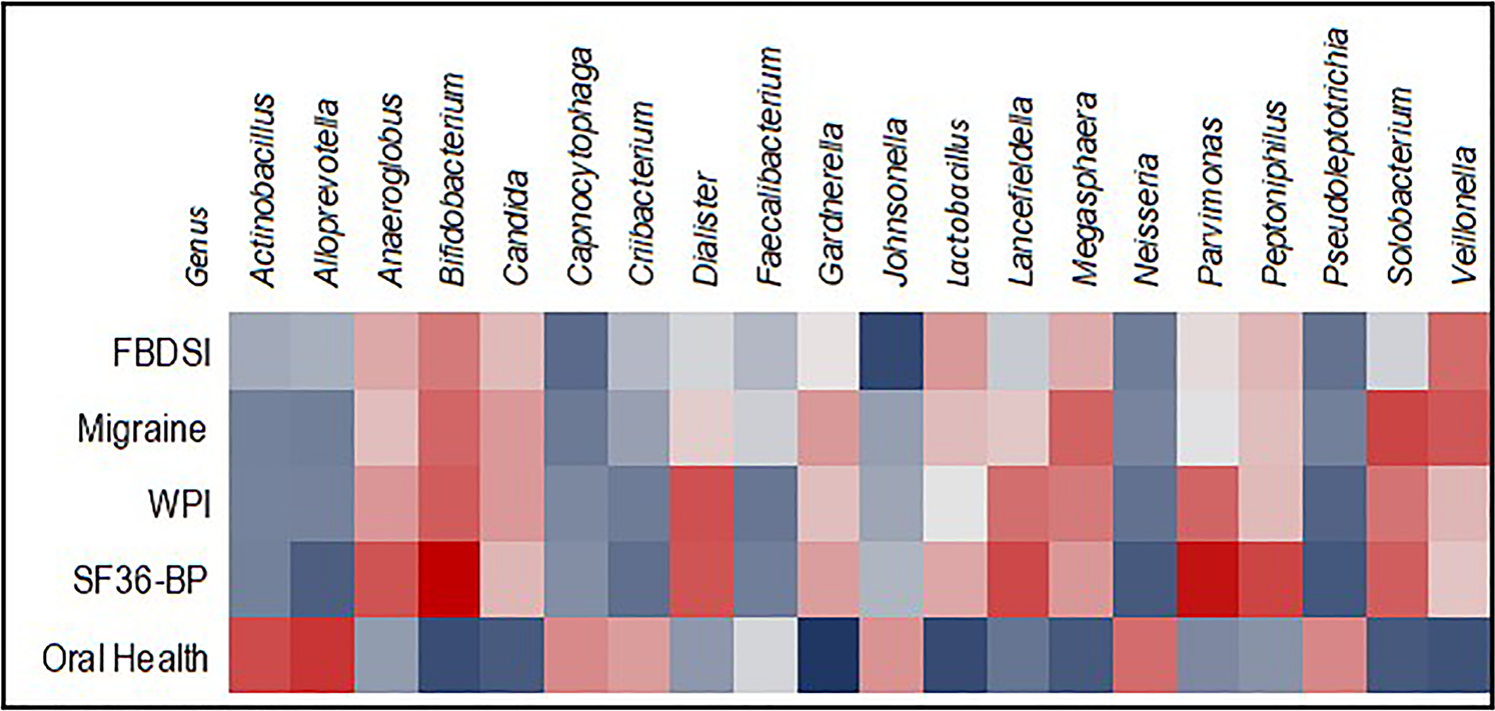
Heatmap showing correlations between relative abundance of 20 genera and oral health, pain, and migraine scores. Inverse correlations are blue, positive correlations are red.

Oral health scores correlated positively with a relative abundance of three genera and inversely with six (*q* < 0.10) and an additional five at *q* = 0.10. The strongest correlation seen was with the genus *Gardnerella* (*ρ* = −0.308), as shown in Figure 6.

### Microbial genera and body pain

After adjusting for confounders in the GLM, associations between SF36-BP and *Anaeroglobus*, *Bifidobacterium*, *Dialister*, *Lancefieldella*, *Parvimonas*, and *Solobacterium* (all with a positive effect on SF36-BP) and *Aggregatibacter*, *Neisseria*, and *Pseudoleptotrichia* all with a negative effect (all *p* < 0.05) were seen. The strongest associations after FDR adjustment were *Parvimonas* (est. 0.02, SE 0.00, *t* = 3.0, *q* = 0.016) and *Solobacterium* (est. 0.01, SE 0.00, *t* = 3.1, *q* = 0.017). A significant association between age and *Neisseria* was seen (est. 0.04, SE 0.02, *t* = 2.5, *p* = 0.02, *q* = 0.41).

Relative abundance of *Dialister* was positively associated with WPI scores, and inverse associations were seen with the genera *Pseudoleptotrichia* and *Solobacterium* (all *p* < 0.05). None were significant after FDR, and no interactions for age, BMI, or added dietary sugar were seen.

Fifteen genera correlated with the SF36-BP and another 15 with the WPI (*p* < 0.05 before applying the FDR), of which 10 were common to both body pain indices at *p* < 0.05. Of those correlated with the SF36-BP score, 10 remained significant after FDR (all *q* < 0.08). None of those associated with the WPI retained significance after applying the Benjamini–Hochberg formula.

The 15 genera with the strongest correlation coefficients from each analysis are included in Table 3, with FDR adjustments. From the amalgamated genera (*n* = 20), six are inversely correlated and 14 positively correlated with the pain scores. The strongest correlations seen with pain were *Bifidobacterium* (*ρ* = 0.319) and *Parvimonas* (*ρ* = 0.300) and the SF36-BP score.

**Table 3:** Correlational analysis between the relative abundance of oral genera and body pain scores.

Oral genera	SF36 bodily pain score (*n* = 156)				Widespread pain index (*n* = 158)			
	Rho	*p-*value	95% CI	*q*	Rho	*p-*value	95% CI	*q*
*Aggregatibacter*	−0.160	0.05	−0.31, 0.00	0.41	−0.107	0.18	−0.26, 0.05	0.63
*Alloprevotella*	−0.222	0.01	−0.37, −0.06	0.07	−0.144	0.07	−0.29, 0.02	0.53
*Anaeroglobus*	0.234	0.003	0.08, 0.38	0.06	0.166	0.04	0.00, 0.31	0.44
*Bifidobacterium*	0.319	<0.001	0.17, 0.46	0.01	0.222	0.005	0.07, 0.37	0.20
*Candida*	0.127	0.12	−0.03, 0.28	0.46	0.158	0.049	0.00, 0.31	0.44
*Criibacterium*	−0.186	0.02	−0.33, −0.03	0.24	−0.161	0.044	−0.31, 0.00	0.39
*Dialister*	0.233	0.003	0.08, 0.38	0.05	0.240	0.003	0.08, 0.38	0.18
*Faecalibacterium*	−0.153	0.06	−0.30, 0.01	0.42	−0.175	0.03	−0.32, −0.01	0.36
*Lactococcus*	−0.077	0.34	−0.23, 0.08	0.74	−0.248	0.002	−0.39, −0.09	0.20
*Lancefieldella*	0.245	0.002	0.09, 0.39	0.07	0.207	0.01	0.05, 0.35	0.21
*Megasphaera*	0.161	0.045	0.00, 0.31	0.44	0.196	0.01	0.03, 0.34	0.22
*Mycoplasma*	0.127	0.11	−0.03, 0.28	0.46	0.190	0.02	0.04, 0.34	0.23
*Neisseria*	−0.235	0.003	−0.38, −0.08	0.07	−0.187	0.02	−0.33, −0.02	0.28
*Parvimonas*	0.303	<0.001	0.15, 0.44	0.01	0.217	0.01	0.06, 0.36	0.22
*Peptoniphilus*	0.248	0.002	0.09, 0.39	0.08	0.125	0.12	−0.03, 0.27	0.51
*Pseudoleptotrichia*	−0.237	0.003	−0.38, −0.08	0.07	−0.212	0.01	−0.36, −0.05	0.19
*Simonsiella*	−0.172	0.03	−0.32, −0.01	0.34	−0.121	0.13	−0.27, 0.04	0.51
*Scardovia*	0.131	0.11	−0.03, 0.28	0.46	0.160	0.045	0.00, 0.31	0.42
*Slackia*	0.158	0.05	0.00, 0.31	0.38	0.069	0.39	−0.09, 0.22	0.76
*Solobacterium*	0.223	0.01	0.06, 0.37	0.07	0.203	0.01	0.05, 0.35	0.22

*q*, adjusted *p*-value after Benjamini–Hochberg correction.

### Microbial genera and migraine

*Johnsonella* showed a negative association with migraine scores in the GLM (est. −1.2, SE 0.53, *t* *=* −2.3, *p* = 0.03), and a positive association was seen for *Mycoplasma* (est. 1.23, SE 0.57, *t* = 2.17, *p* = 0.04). None were significant after adjustment for FDR, and no significant association was seen for age, BMI, or added dietary sugar.

A comparison between women with and without migraine, showing the relative abundance of genera that were correlated with migraine as detailed above is shown in Table 4. A visual comparison of these genera, with mean read counts, is shown in Supplementary Figure S3.

**Table 4:** Differences in relative abundance of oral genera in women (*n* = 156) with and without migraine.

Genus	No migraine (*n* = 80)		Migraine (*n* = 76)		Mann–Whitney *U*	
	Mean (*SD*)	Median (IQR)	Mean (*SD*)	Median (IQR)	*Z*	*p*-value
*Bacteroides*	0.27 (1.5)	0.27 (1.7)	0.01 (1.5)	0.1 (1.8)	−1.280	0.201
*Bifidobacterium*	−2.73 (2.2)	−3.86 (2.6)	−1.88 (2.5)	−2.81 (3.5)	−2.709	0.007
*Capnocytophaga*	5.90 (1.0)	6.03 (1.3)	5.54 (0.9)	5.44 (1.3)	−2.812	0.005
*Dialister*	3.62 (1.0)	3.65 (1.5)	3.59 (1.3)	3.73 (1.6)	−0.475	0.635
*Johnsonella*	3.74 (1.1)	3.92 (1.1)	2.34 (2.5)	3.03 (3.0)	−3.828	<0.001
*Lactobacillus*	−1.38 (2.2)	−1.22 (3.7)	−0.84 (2.3)	−0.74 (3.3)	−1.518	0.129
*Pseudoleptotrichia*	0.87 (2.7)	1.64 (3.0)	−0.08 (2.8)	1.18 (5.7)	−2.064	0.04
*Mycoplasma*	0.71 (2.1)	1.01 (2.8)	1.72 (2.1)	2.13 (2.2)	−3.492	<0.001
*Odoribacter*	−2.82 (1.6)	−3.56 (2.6)	−3.34 (1.5)	−3.96 (1.0)	−2.138	0.03
*Veillonella*	8.18 (0.7)	8.21 (0.8)	8.42 (0.7)	8.37 (0.9)	−1.766	0.08

Migraine scores were weakly correlated with the relative abundance of nine genera. Positive correlations were observed for *Bifidobacterium* [*ρ* = 0.189, *p* = 0.02, 95% CI (0.03, 0.34)], *Dialister* [*ρ* = 0.243, *p* = 0.003, 95% CI (0.05, 0.38)], *Lactobacillus* [*ρ* = 0.157, *p* = 0.05, 95% CI (−0.00, 0.31)], *Mycoplasma* [*ρ* = 0.259, *p* = 0.001, 95% CI (0.10, 0.40)], and *Veillonella* [*ρ* = 0.206, *p* = 0.01, 95% CI (0.05, 0.35)]. Inverse correlations were seen with *Bacteroides* [*ρ* = −0.162, *p* = 0.04, 95% CI (−0.31, −0.0)], *Capnocytophaga* [*ρ* = −0.195, *p* = 0.01, 95% CI (−0.416, −0.11)], *Johnsonella* [*ρ* = −0.268, *p* < 0.001, 95% CI (−0.41, −0.11)], *Odoribacter* [*ρ* = −0.159, *p* = 0.049, 95% CI (−0.31, 0.00)], and *Pseudoleptotrichia* [*ρ* = −0.177, *p* = 0.03, 95% CI (−0.33, −0.02)]. After applying the BH for FDR, two remained significant: *Johnsonella* (*q* = 0.08) and *Mycoplasma* (*q* = 0.06).

For migraine type, the relative abundance of *Dialister*, *Lancefieldella*, *Megasphaera*, and *Selemonas* was positively associated with all three migraine types and *Atopodium* and *Veillonella* with frequent and chronic migraine. *Lancefieldella* remained significantly positively associated with all migraine types after FDR (*p* ≤ 0.10), as follows: frequent, est. 6.27, SE 1.7, *t* = 3.7, *q* = 0.08, chronic, est. 6.59, SE 1.7, *t* = 3.8, *q* = 0.09; and infrequent, est. 6.72, SE 1.7, *t* = 3.9, *q* = 0.07. No significant associations were seen for the confounders in this model.

A Kruskal–Wallis *H* test showed that migraine type was significantly associated with relative abundance of *Mycoplasma* [*H* = 16.4 (3), *p* < 0.001] and *Johnsonella* [*H* = 16.40 (3), *p* < 0.001]. For both genera, frequent migraine vs. no migraine was (*p* < 0.001 and *p* = 0.01, respectively) after Bonferroni correction. These were not significant in the regression model.

### Microbial genera and abdominal pain

Results from the correlational analysis between the relative abundance of oral genera and abdominal pain as measured by the FBDSI are shown in Table 5. Six of these genera were also correlated with at least one of the three other pain measures used. None were statistically significant after FDR adjustment.

**Table 5:** Correlations between relative abundance of oral genera and functional bowel disorder severity scores in 156 women.

Genus	Rho	*p*-value	95%CI	*q*
*Bifidobacterium*	0.211	0.01	0.05, 0.36	0.20
*Candida*	0.158	0.049	0.00, 0.31	0.73
*Capnocytophaga*	−0.160	0.046	−0.31, −0.00	0.79
*Gardnerella*	0.157	0.05	−0.00, 0.31	0.67
*Megasphaera*	0.214	0.007	0.06, 0.36	0.22
*Prevotella*	0.239	0.003	0.08, 0.38	0.18
*Solobacterium*	0.246	0.002	0.09, 0.39	0.24
*Stomatobaculum*	0.157	0.05	−0.0, 0.31	0.60
*Veillonella*	0.228	0.004	0.07, 0.37	0.16

*q*, adjusted *p*-value after Benjamini–Hochberg correction.

### Microbial phyla and oral health

Five key phyla made up 99.6% of the oral microbiome (Supplementary Figure S2).

The strongest associations with phyla and OH score in the GLM were seen for Candidatus Saccharibacteria (est. 0.04, SE 0.02, *t* = 2.7, *p* = 0.01, *q* = 0.12) and Ascomycota (est. −0.04, SE 0.06, *t* = −2.5, *p* = 0.02, *q* = 0.13). No significant relationships were observed for age or BMI.

A weak inverse correlation was seen for OH score and the relative abundance of the fungal phylum Ascomycota [*ρ* = −0.220, *p* = 0.006, 95% CI (−0.36, −0.06), *q* = 0.08]. The strongest positive correlation was between OH and the phylum *Proteobacteria* [*ρ* = 0.171, *p* = 0.03, 95% CI (0.01, 0.32), *q* = 0.23].

### Microbial phyla and body pain

Examining the GLM for interaction between phyla and the WPI and SF36-BP, weak positive effects were seen for age on the relative abundance of *Proteobacteria* (*p* < 0.05) in both models and a weak inverse effect of added dietary sugar on the relative abundance of *Mycoplasmatota* (*p* < 0.04) also in both models. None were significant after FDR.

When evaluating the relative abundance of oral phyla against measures of body pain, *Mycoplasmatota* (formerly *Tenericutes*) was weakly correlated with the WPI [*ρ* = 0.183, *p* = 0.02, 95% CI (0.03, 0.33), *q* = 0.15].

The SF36-BP score was inversely correlated with *Proteobacteria* [*ρ* = −0.174, *p* = 0.03, 95% CI (−0.32, −0.02), *q* = 0.40].

### Microbial phyla and migraine

Relative abundance of the phylum *Mycoplasmatota* was positively correlated with migraine scores [*ρ* = 0.254, *p* = 0.001, 95% CI (0.1, 0.4), *q* = 0.02] and was significantly higher in women with migraine [Md (IQR) −0.3 (2.3)] compared to those without migraine [−1.3 (2.3), *Z* *=* −2.0, *p* < 0.001, *q* = 0.01]. Adjusting for the expected influence of BMI, age, and added dietary sugar in the GLM model, this relationship was insignificant (*p* = 0.07).

The relative abundance of Candidatus Saccharibacteria was lower in migraineurs than those without migraine [Md (IQR) 0.07 (2.6) cf. 1.4 (1.6), *Z* *=* −2.8, *p* = 0.005, *q* = 0.04] and was weakly inversely correlated with migraine scores [*ρ* = −0.183, *p* = 0.02, 95% CI (−0.3, −0.3), *q* = 0.15]. In the GLM, this phylum was associated with migraine after adjusting for BMI, age, and added dietary sugar (est. −1.3, *SE* 0.5, *t* = 2.6, *p* = 0.01, *q* = 0.16).

The phylum *Proteobacteria* was insignificantly lower in migraineurs [Md (IQR) 5.9 (1.0), cf. 6.1 (0.7), *Z* = −2.0, *p* = 0.05] and weakly inversely correlated with migraine scores [*ρ* = −0.176, *p* = 0.03, 95% CI (−0.3, −0.2), *q* = 0.13]. In the GLM, the relative abundance of *Proteobacteria* was significantly associated with age before FDR (est. 0.02, SE 0.2, *t* = −0.3, *p* = 0.04), and no association was observed between migraine and *Proteobacteria* (*p* = 0.75).

### Microbial phyla and abdominal pain

An association was seen between *Fusobacteria* and the FBDSI score in the regression model [est. 0.20, SE 0.09, *p* = 0.02, *q* = 0.22, 95% CI (0.036, 0.370)]. No significant associations were seen between severity groupings of FBD and the relative abundance of any of these 14 phyla (Kruskal–Wallis *H* test). No significant correlations were seen between oral phyla and FBDSI scores.

### The oral microbial diversity metrics and oral health

KO richness was weakly correlated (*p* = 0.045) with the total OH score. No other correlations were observed between OH and indices of oral species richness and diversity.

The Shannon index was weakly but significantly inversely correlated with scores for migraine [*ρ* = −0.242, *p* = 0.003, 95% CI (−0.39, −0.08)] and FBD severity [*ρ* = −0.175, *p* = 0.03, 95% CI (−0.33, −0.02)]. No other significant relationships were seen between the WPI or SF36-BP scores and species richness, KO richness, or the Shannon diversity index (all *p* > 0.49).

## Discussion

We explored the relationship between OH, the oral microbiome, and pain associated with central sensitisation disorders, migraine, fibromyalgia, and functional bowel pain. Lower OH scores correlated with higher scores for each of these measures of pain.

Common themes were seen between OH scores, pain, and known, or potential oral pathogens. Four species that were significantly associated with body pain and poor OH include *Solobacterium moorei*, an anaerobic gram-positive pathogen that produces volatile compounds strongly associated with halitosis and promotes the coexistence of *Porphyromonas* and *Prevotella* in biofilm (27); *Dialister pneumosintes*, a gram-negative anaerobic periodontal pathogen, linked to extraoral infections, including cerebral and neck/mediastinal abscesses (28); *Fusobacterium nucleatum*, a known gram-negative oral pathogen (29); and *Parvimonas micra*, a gram-positive opportunistic pathogen, recently identified as a biomarker for colorectal cancer (30). *Gardnerella vaginalis*, an opportunistic pathobiont of the vaginal microbiota, the growth of which is promoted by the presence of *F. nucleatum* (31), was also associated with poor OH. We did not collect genitourinary health data from this all-female cohort.

*Mycoplasma* (phyla: *Mycoplasmatota*) was implicated in migraine and migraine type at each taxonomical level. *M. salivarium* is a common oral resident, though not generally considered pathogenic in immunocompetent people. However, it is a putative player in periodontal disease (32), with a demonstrable ability to stimulate fibroblasts in the gingiva and peripheral mononuclear cells and upregulate inflammatory cytokines. *M. salivarium* has been isolated from the synovium of patients with TMJ pain and may have a causative role (33).

The prevalence of *Bifidobacterium* at a genus level was significantly associated with OH, body pain, and migraine, with relative abundance inversely associated with OH scores, and positively associated with pain, raising questions over its status as a probiotic in this context. Considered to be of health benefit, *Bifidobacteriaceae* are anaerobes and generally reside in the distal gastrointestinal tract but are acid-producing, acid-resistant and somewhat fluoride-resistant, so they are less impacted by toothbrushing. In dentate people, *B*. *dentium* is the most prevalent *Bifidobacterium* species in the mouth and has been associated with dental caries (34).

Consideration of the role of the human microbiome in pain physiology is relatively novel. Several recent reviews have explored theories of microbe-driven mechanisms in neuropathic and general pain (35–38). Noteworthy is the often-insidious onset of chronic pain, making triggers challenging to identify. Changes in neural activity have been observed up to 12 months before the onset of nociplastic musculoskeletal, headache, and abdominal pain in children (4), which to date has not been examined in the context of OH.

The human microbiota produces, metabolises, and induces a vast array of substances, many of which are candidates of pain signalling. For example, bacterial activity can induce substance P, calcitonin gene-regulated peptide (CGRP) (39), and vascular endothelial growth factor (VEGF). Bacteria can also alter DNA methylation (40) and affect the balance of glutamate and γ-aminobutyric acid (GABA)—all possible contributors to increased sensitivity in models of peripheral, neuropathic, and/or visceral pain (38, 39, 41). CGRP, found in cutaneous tissue and sensory nerves (42), is inducible by bacterial lipopolysaccharide (LPS) (43). Prolonged exposure to CGRP, a strong vasodilator, causes increased peripheral sensitisation, likely due to the induction of inflammatory cytokines from immune cells and nerve endings (44). Antagonising CGRP is an effective measure to reduce neuropathic pain (45) and alleviate migraine (39), indicating the potential for addressing migraine from the perspective of microbiome-directed therapy.

VEGF, a potent stimulator of angiogenesis, contributes to neuroinflammation and visceral hypersensitivity and is believed to be an important chemokine in the development of nociceptive sensitisation (46). Elevations in VEGF abundance have been found at higher concentrations in the blood of people suffering migraine (47), fibromyalgia (48), and periodontal disease (49).

Several gram-negative bacteria were differentially abundant across the various measures of pain in this cohort. LPS, from the cell walls of gram-negative bacteria, is an endotoxin, which influences immune responses (50) and is implicated in the induction of pro-inflammatory cytokines in CRPS and fibromyalgia, both central sensitisation disorders (43). Translocation of LPS to the systemic circulation may be increased when tight cell junctions are compromised (51), noting that cellular hyperpermeability is not limited to gut epithelium (52).

Specific periodontal pathogens aside, little is known about the connections between OH and general health, with precise mechanisms an area of active investigation (12). Noteworthy is our observation of higher prevalence and abundance of commensal oral microbial inhabitants in those with higher OH and lower pain scores, supporting an opposite direction of effect between microbial commensals and pathogens and pain.

Genus-level results differ from those in a study by Jiang et al. (53), in which higher relative abundances of 15 genera, as measured by 16S rRNA, were associated with migraine. Consistent with their results was a higher abundance of the genus *Mycoplasma* in our migraineurs (which was also correlated with migraine scores). No other congruencies were found between the two studies, likely due to their smaller cohort (*n* = 26) and the different sequencing and data analysis techniques employed.

There are no well-defined criteria for a healthy oral microbiome. Rather, our understanding has been largely informed by the Human Oral Microbiome Database, an initiative of the human microbiome project (10), and investigations into bacteria that cause oral diseases. The mouth is expected to harbour a range of pathobionts and commensals. A primary role of the latter is protection against pathobiont overgrowth via a range of mechanisms, including competitive inhibition and production of secretory IgA and antimicrobial peptides (54).

The notion that the health of the mouth and the microbes therein may be important in the pathogenesis of extraoral conditions has attracted increased attention. It is entirely plausible that poor OH, leading to gum disease and oral dysbiosis, sets the stage for translocation of bacteria and microbial metabolites directly to the systemic venous and lymphatic circulation. We hypothesise that these metabolites and/or bacteria trigger heightened pain signalling and defects in pain mechanisms, contributing to the pathogenesis of idiopathic nociplastic pain that is associated with these central sensitisation disorders.

Therefore, from a clinical perspective, studies are warranted to determine whether improving OH and promoting eubiosis reduces the pain of central sensitisation disorders. Importantly, from an epidemiological perspective, our findings contribute to the knowledge pool, implicating OH as a modifiable factor in the aetiology of chronic pain conditions. Public health initiatives that raise awareness and support preventive OH measures are indicated.

### Limitations and strengths

There are several limitations to consider in the context of our results. While the possibility exists that pain itself may initiate alterations to the microbiota, there is no evidence of this to date. Limited comparability to other research is expected due to differing statistical programmes (22) and microbiome bioinformatic tools employed. Our data were obtained using RNA metatranscriptomics, which quantifies microbial gene expression and provides a more extensive dataset, but is not directly comparable to 16s rRNA or 16sDNA. An inherent confounder when making multiple comparisons is the severity of corrections to minimise type 1 error, compared to smaller datasets. Thus, our results are at risk of a type II error. Considering this, clinically relevant results, if not always meeting rigorous statistical thresholds, are presented.

Oral health scores were based on self-reported measures. Our participants did not undergo oral examination; thus, beyond self-report, we do not know if periodontal disease was present. While the WHO's OH survey has no formal scoring algorithm, it extensively interrogates oral hygiene practices, dental health history, and specific dietary and lifestyle habits known to affect OH.

The need for this analysis was identified during a larger observational study (15); thus, cause and effect cannot be extrapolated. The pain assessments used were not limited to one measure, adding to the validity of our findings. Migraine and fibromyalgia are commonly comorbid (8), limiting the generalisability of these results to all migraine sufferers.

Saliva is expected to provide a reasonable representation of the overall oral microbial community but may not be comparable to data obtained from specific oral sites.

## Conclusion

The chronicity of central sensitisation pain is a debilitating phenomenon that negatively impacts the quality of life. Without identifiable pathophysiology, any idiopathic pain poses notable challenges for clinicians and patients alike.

We report lower OH scores and higher abundance of a range of oral pathobionts to be associated with higher pain scores, suggesting a contribution to the pain phenotype. Subject to larger studies confirming these findings, we propose that the oral microbiome has a role in pain signalling and requires consideration of an oral microbiome–nervous system axis.

## Data Availability

The original contributions presented in the study are included in the article and/or the Supplementary Material, further inquiries can be directed to the corresponding author.
