# Supplementary material for: An association between poor oral health, oral microbiota, and pain identified in New Zealand women with central sensitisation disorders: a prospective clinical study
Source: Front Pain Res (Lausanne). 2025 Apr 9;6:1577193. doi: 10.3389/fpain.2025.1577193 (PMC12014678; doi:10.3389/fpain.2025.1577193)
Supplement: Supplementary file 1 [file Table1.docx]

| **Max** | **Min** | **ORAL HEALTH QUESTIONNAIRE FOR ADULTS (WHO) c (**[details omitted for double-anonymized peer review]. **[unpublished]** | | | | | | | | | | | | |
| --- | --- | --- | --- | --- | --- | --- | --- | --- | --- | --- | --- | --- | --- | --- |
| 60 | -2 | **Domain 1: ORAL HEALTH HISTORY & PHYSICAL STATE OF TEETH** | | | | | | | | | | | | |
|  |  | How many natural teeth do you have? | **None** | | **1-9** | | **10 - 19** | | **20 or more** | |  | |  |  |
|  |  |  | 0 | | 1 | | 2 | | 3 | |  | |  |  |
|  |  | Do you have removable dentures? | **Yes** | | **No** | |  | |  | |  | |  |  |
|  |  |  | 0 | | 1 | |  | |  | |  | |  |  |
|  |  | What type of removal denture do you have? (Multiple choices allowed) | **Partial denture** | | **Full upper denture** | | **Full lower denture** | |  | |  | |  |  |
|  |  |  | -1 | | -1 | | -1 | |  | |  | |  |  |
|  |  | **Please indicate whether you have had any of the following dental work:** | **Don't know** | | **Yes / Current** | | **In the past, now removed** | | **No** | |  | |  |  |
|  |  | Root canals | 0 | | 0 | | 1 | | 2 | |  | |  |  |
|  |  | Caps or crowns | 0 | | 0 | | 1 | | 2 | |  | |  |  |
|  |  | Dental implants | 0 | | 0 | | 1 | | 2 | |  | |  |  |
|  |  | Other orthodontic work | 0 | | 0 | | 1 | | 2 | |  | |  |  |
|  |  | **How many:** | **5 or more** | | **Four** | | **Three** | | **Two** | | **One** | |  |  |
|  |  | How many root canal filled teeth do you have? | 0 | | 1 | | 2 | | 3 | | 4 | |  |  |
|  |  | How many caps or crowns do you have? | 0 | | 1 | | 2 | | 3 | | 4 | |  |  |
|  |  | How many dental implants do you have? | 0 | | 1 | | 2 | | 3 | | 4 | |  |  |
|  |  | **Pain** | **Yes** | | **Don't know** | | **No** | |  | |  | |  |  |
|  |  | During the past 12 months, did your teeth or mouth cause you any pain or discomfort? | 0 | | 0 | | 4 | |  | |  | |  |  |
| 48 | 0 | **Domain 2: ORAL HEALTH PROBLEMS** | | | | | | | | | | | | |
|  |  | **What other oral health problem or problems have you had?** | | **Current problem** | | **Recent problem (in the last 3m)** | | **Recent problem (3 - 12m ago)** | | **Past problem (resolved > 12m ago)** | | **Never had this problem** | | |
|  |  | Constant pain in 1 or more teeth | | 0 | | 1 | | 2 | | 3 | | 4 | | |
|  |  | Transient pain in 1 or more teeth | | 0 | | 1 | | 2 | | 3 | | 4 | | |
|  |  | Pain in the gums | | 0 | | 1 | | 2 | | 3 | | 4 | | |
|  |  | Bleeding gums when brushing | | 0 | | 1 | | 2 | | 3 | | 4 | | |
|  |  | Bleeding gums when eating | | 0 | | 1 | | 2 | | 3 | | 4 | | |
|  |  | Abcess or infection in 1 or more teeth | | 0 | | 1 | | 2 | | 3 | | 4 | | |
|  |  | Bad breath | | 0 | | 1 | | 2 | | 3 | | 4 | | |
|  |  | Tooth sensitivity to cold | | 0 | | 1 | | 2 | | 3 | | 4 | | |
|  |  | Tooth staining | | 0 | | 1 | | 2 | | 3 | | 4 | | |
|  |  | Braces/retainer | | 0 | | 1 | | 2 | | 3 | | 4 | | |
|  |  | Dry mouth | | 0 | | 1 | | 2 | | 3 | | 4 | | |
|  |  | Other oral health problem | | 0 | | 1 | | 2 | | 3 | | 4 | | |

| 22 | 0 | **Domain 3: ORAL CARE (Preventive Behaviour)** | | | | | | | | |
| --- | --- | --- | --- | --- | --- | --- | --- | --- | --- | --- |
|  |  | How often do you clean your teeth? | **Never** | **Once a month** | **2-3 times a month** | **Once a week** | **2-6 times a week** | **Once a day** | **Twice or more a day** |  |
|  |  |  | 0 | 1 | 2 | 3 | 4 | 5 | 6 |  |
|  |  | Do you use any of the following to clean your teeth? (Multiple choices allowed) | **Toothpaste** | **Toothbrush** | **Wooden toothpicks** | **Plastic toothpicks** | **Dental floss** | **Interdental brushes** | **Mouthrinse** | **Other** |
|  |  |  | 1 | 1 | 1 | 1 | 1 | 1 | 1 | 1 |
|  |  | What else do you use to clean your teeth? (Please tell us what you use): | | |  |  |  |  |  |  |
|  |  | Do you use a flouridated toothpaste? | **Don't know** | **No** | **Yes** |  |  |  |  |  |
|  |  |  | 0 | 0 | 1 |  |  |  |  |  |
|  |  | How long is it since you last saw a dentist or dental hygienist? | **Never received dental care** | **More than 5 y ago** | **Between 2 -5 y ago** | **Between 1- 2y ago** | **6-12m ago** | **Less than 6m ago** |  |  |
|  |  |  | 0 | 1 | 2 | 3 | 4 | 5 |  |  |
|  |  | What was the reason of your last visit to the dentist or dental hygienist? | **Don't know or can't remember** | **Pain or trouble with teeth, mouth or gums** | **Treatment or follow-up treatment** | **Consultation/advice** | **Routine check-up/treatment** |  |  |  |
|  |  |  | 0 | 0 | 1 | 2 | 3 |  |  |  |
| 12 | 0 | **Domain 4: ORAL HEALTH AFFECTS ORAL FUNCTION** | | | | | | | | |
|  |  | **Because of the state of your teeth or mouth, how often have you experienced any of the following problems during the past 12 months?** | **Don't know** | **Very often** | **Fairly often** | **Sometimes** | **No** |  |  |  |
|  |  | Difficulty in biting foods | 0 | 0 | 1 | 2 | 3 |  |  |  |
|  |  | Difficulty chewing foods | 0 | 0 | 1 | 2 | 3 |  |  |  |
|  |  | Difficulty with speech/trouble pronouncing words | 0 | 0 | 1 | 2 | 3 |  |  |  |
|  |  | Dry mouth | 0 | 0 | 1 | 2 | 3 |  |  |  |
| 24 | 0 | **Domain 5: ORAL HEALTH AFFECTS PSYCHO-SOCIAL WELL-BEING** | | | | | | | | |
|  |  | Felt embarrassed due to appearance of teeth | 0 | 1 | 2 | 3 | 0 |  |  |  |
|  |  | Felt tense because of problems with teeth or mouth | 0 | 1 | 2 | 3 | 0 |  |  |  |
|  |  | Have avoided smiling because of teeth | 0 | 1 | 2 | 3 | 0 |  |  |  |
|  |  | Had sleep that is often interuppted because of teeth/mouth | 0 | 1 | 2 | 3 | 0 |  |  |  |
|  |  | Have taken days off work because of teeth/mouth | 0 | 1 | 2 | 3 | 0 |  |  |  |
|  |  | Difficulty doing usual activities because of teeth/mouth | 0 | 1 | 2 | 3 | 0 |  |  |  |
|  |  | Felt less tolerant of spouse or people who are close to you | 0 | 1 | 2 | 3 | 0 |  |  |  |
|  |  | Have reduced participation in social activities because of teeth/mouth | 0 | 1 | 2 | 3 | 0 |  |  |  |

| 96 | 0 | **Domain 6: DIET & LIFESTYLE INFLUENCES on ORAL HEALTH** | | | | | | | | | |
| --- | --- | --- | --- | --- | --- | --- | --- | --- | --- | --- | --- |
| *(56)* | 0 | **Diet** | | | | | | | | | |
|  |  | **How often do you eat or drink any of the following foods, even in small quantities?** |  |  |  |  |  |  |  | |  |
|  |  | Fresh fruit | **Seldom or never** | **Several times a month** | **Once a week** | **Several times a week** | **Every day** | **Several times a day** |  |  | |
|  |  |  | 0 | 1 | 2 | 3 | 4 | 5 |  | |  |
|  |  |  | **Several times a day** | **Every day** | **Several times a week** | **Once a week** | **Several times a month** | **Seldom or never** |  | |  |
|  |  | Biscuits, cakes, cream cakes | 0 | 1 | 2 | 3 | 4 | 5 |  | |  |
|  |  | Sweet pies, buns | 0 | 1 | 2 | 3 | 4 | 5 |  | |  |
|  |  | Jam or honey | 0 | 1 | 2 | 3 | 4 | 5 |  | |  |
|  |  | Chewing gum | 0 | 1 | 2 | 3 | 4 | 5 |  | |  |
|  |  | Sweets or chocolates | 0 | 1 | 2 | 3 | 4 | 5 |  | |  |
|  |  | Lemonade, Coca-cola or other soft drinks | 0 | 1 | 2 | 3 | 4 | 5 |  | |  |
|  |  | Tea with sugar | 0 | 1 | 2 | 3 | 4 | 5 |  | |  |
|  |  | Coffee with sugar | 0 | 1 | 2 | 3 | 4 | 5 |  | |  |
|  |  | Kombucha | 0 | 1 | 2 | 3 | 4 | 5 |  | |  |
|  |  | Other sweetened drink | 0 | 1 | 2 | 3 | 4 | 5 |  | |  |
|  |  | Is the chewing gum you use sugarless? | **Don't Know** | **No** | **Yes** |  |  |  |  | |  |
|  |  |  | 0 | 0 | 1 |  |  |  |  | |  |
| *(35)* | 0 | **Tobacco Use** | **Every day** | **Several times a week** | **Once a week** | **Several times a month** | **Seldom** | **Never** |  | |  |
|  |  | Do you smoke or use chewing tobacco? | 0 | 1 | 2 | 3 | 4 | 5 |  | |  |
|  |  | How often do you use cigarettes? | 0 | 1 | 2 | 3 | 4 | 5 |  | |  |
|  |  | How often do you use cigars? | 0 | 1 | 2 | 3 | 4 | 5 |  | |  |
|  |  | How often do you use a pipe? | 0 | 1 | 2 | 3 | 4 | 5 |  | |  |
|  |  | How often do you use chewing tobacco? | 0 | 1 | 2 | 3 | 4 | 5 |  | |  |
|  |  | How often do you use snuff? | 0 | 1 | 2 | 3 | 4 | 5 |  | |  |
|  |  | How often do you use other type/s of tobacco? | 0 | 1 | 2 | 3 | 4 | 5 |  | |  |
|  |  | Please specify the type of tobacco you use: |  |  |  |  |  |  |  | |  |
|  |  | **Alcohol** | **5 or more drinks** | **4 drinks** | **3 drinks** | **2 drinks** | **1 drink** | **None, or < 1 drink** |  | |  |
| (5) | 0 | During the past 30 days, on the days you drank alcohol, how many drinks did you usually drink per day? (1 drink is 300mL beer, 100mL wine or 30mL spirits) | 0 | 1 | 2 | 3 | 4 | 5 |  | |  |
| 10 | 0 | **Domain 7: SELF-AWARENESS OF PROBLEMS (Locker's 2-questions)** | | | | | | | | | |
|  |  | ***How would you describe:*** | **Don't know** | **Very poor** | **Poor** | **Average** | **Good** | **Very good** | **Excellent** | | |
|  |  | - the state of your teeth? | 0 | 0 | 1 | 2 | 3 | 4 | 5 | | |
|  |  | - the state of your gums? | 0 | 0 | 1 | 2 | 3 | 4 | 5 | | |
|  |  | *Ref: Petersen, P. E., Baez, R. J., & World Health Organization. (2013). Oral Health Surveys: Basic Methods (5 ed. Vol. vii). Geneva: World Health Organisation* | | | | | | | | | |
