# Supplementary material for: An association between poor oral health, oral microbiota, and pain identified in New Zealand women with central sensitisation disorders: a prospective clinical study
Source: Front Pain Res (Lausanne). 2025 Apr 9;6:1577193. doi: 10.3389/fpain.2025.1577193 (PMC12014678; doi:10.3389/fpain.2025.1577193)
Supplement: Supplementary file 2 [file Table2.docx]

|  | Oral Health Score | | |
| --- | --- | --- | --- |
|  | **Rho** | **95% CI** | ***P*-value** |
| Short form 36 bodily pain (inverted score) | -0.552 | -0.66, -0.43 | <.001 |
| Widespread pain index | -0.555 | -0.66, -0.43 | <.001 |
| Migraine symptom score | -0.388 | -0.52, -0.241 | <.001 |
| Functional bowel disorder severity index | -0.475 | -0.59, -0.34 | <.001 |
