# Supplementary material for: An association between poor oral health, oral microbiota, and pain identified in New Zealand women with central sensitisation disorders: a prospective clinical study
Source: Front Pain Res (Lausanne). 2025 Apr 9;6:1577193. doi: 10.3389/fpain.2025.1577193 (PMC12014678; doi:10.3389/fpain.2025.1577193)
Supplement: Supplementary file 3 [file Table3.docx]

| Oral Species | Rho | *p*-value | 95% CI | Adj *p*-value |
| --- | --- | --- | --- | --- |
| *Bifidobacterium dentium* | -0.325 | <.001 | 0.19, 0.48 | .003 |
| *Candida albicans* | -0.303 | <.001 | -0.46, -0.17 | .01 |
| *Fusobacterium massiliense* | 0.255 | .001 | -0.35, -0.04 | .03 |
| *Fusobacterium nucleatum* | -0.259 | .001 | -0.42, -0.13 | .03 |
| *Gardnerella vaginalis* | -0.371 | <.001 | -0.50, -0.22 | .001 |
| *Haemophilus paraphrohaemolyticus* | 0.266 | .001 | 0.04, 0.35 | .02 |
| *Haemophilus pittmaniae* | 0.244 | .002 | -0.35, -0.04 | .04 |
| *Haemophilus sputorum* | 0.344 | <.001 | -0.34, -0.03 | .002 |
| *Kingella oralis* | -0.230 | .004 | 0.10, 0.40 | .048 |
| *Lactobacillus gasseri* | -0.232 | .004 | -0.40, -0.10 | .046 |
| *Lactobacillus paragasseri* | -0.280 | <.001 | -0.44, -0.15 | .02 |
| *Leptotrichia hofstadii* | -0.237 | .003 | 0.11, 0.41 | .045 |
| *Ligilactobacillus salivarius* | -0.234 | .003 | -0.40, -0.11 | .047 |
| *Neisseria cinerea* | 0.259 | .001 | -0.35, -0.04 | .02 |
| *Neisseria flavescens* | 0.233 | .003 | 0.04, 0.35 | .045 |
| *Neisseria polysaccharea* | 0.295 | <.001 | -0.35, -0.04 | .01 |
| *Neisseria subflava* | 0.275 | .001 | 0.04, 0.35 | .02 |
| *Simonsiella muelleri* | 0.242 | .002 | -0.35, -0.04 | .04 |
| *Stomatobaculum longum* | -0.261 | .001 | 0.13, 0.43 | .03 |
| *Streptococcus gallolyticus* | -0.257 | .001 | 0.12, 0.42 | .02 |
| *Streptococcus sp. A12* | 0.234 | .003 | -0.35, -0.04 | .046 |
| *Streptococcus sp. LPB0220* | -0.249 | .002 | -0.41, -0.12 | .03 |
| *Streptococcus timonensis* | 0.286 | <.001 | 0.04, 0.35 | .01 |
| *Veillonella dispar* | -0.270 | .001 | 0.14, 0.43 | .02 |
