# Supplementary material for: An association between poor oral health, oral microbiota, and pain identified in New Zealand women with central sensitisation disorders: a prospective clinical study
Source: Front Pain Res (Lausanne). 2025 Apr 9;6:1577193. doi: 10.3389/fpain.2025.1577193 (PMC12014678; doi:10.3389/fpain.2025.1577193)
Supplement: Supplementary file 4 [file Table4.docx]

| Oral Species | OH *est.* (*SE*) | *t-*statistic | *p*-value | Adj *p-value* |
| --- | --- | --- | --- | --- |
| *Fusobacterium massiliense* | 0.08 (0.02) | 3.77 | .001 | .03 |
| *Fusobacterium nucleatum* | -0.02 (0.01) | -3.26 | .001 | .06 |
| *Gardnerella vaginalis* | -0.09 (0.02) | -4.24 | .001 | .03 |
| *Haemophilus haemolyticus* | 0.04 (0.01) | 2.92 | .005 | .10 |
| *Haemophilus parahaemolyticus* | 0.07 (0.02) | 2.95 | .005 | .10 |
| *Haemophilus paraphrohaemolyticus* | 0.07 (0.02) | 3.25 | .003 | .07 |
| *Haemophilus sputorum* | 0.10 (0.02) | 4.31 | <.001 | .01 |
| *Lactobacillus paragasseri* | -0.07 (0.02) | -3.42 | .006 | .10 |
| *Lancefieldella parvula* | -0.03 (0.01) | -3.07 | .004 | .10 |
| *Leptotrichia hongkongensis* | -0.04 (0.01) | -3.31 | .004 | .06 |
| *Neisseria cinerea* | 0.06 (0.02) | 3.20 | .003 | .08 |
| *Neisseria polysaccharea* | 0.06 (0.02) | 3.36 | .004 | .07 |
| *Neisseria subflava* | 0.07 (0.02) | 3.91 | <.001 | .02 |
| *Simonsiella muelleri* | 0.10 (0.03) | 3.33 | .002 | .06 |
| *Stomatobaculum longum* | -0.05 (0.01) | -3.46 | .001 | .05 |
| *Streptococcus sp. LPB0220* | -0.03 (0.01) | -3.21 | .002 | .06 |
| *Streptococcus timonensis* | 0.03 (0.01) | 3.36 | .001 | .05 |
| *Tannerella sp. oral taxon HOT-286* | 0.03 (0.01) | 3.04 | .003 | .08 |
| *Veillonella dispar* | -0.03 (0.01) | -3.29 | .001 | .06 |
| *Veillonella parvula* | -0.03 (0.01) | -3.39 | .001 | .05 |
