# Supplementary material for: An association between poor oral health, oral microbiota, and pain identified in New Zealand women with central sensitisation disorders: a prospective clinical study
Source: Front Pain Res (Lausanne). 2025 Apr 9;6:1577193. doi: 10.3389/fpain.2025.1577193 (PMC12014678; doi:10.3389/fpain.2025.1577193)
Supplement: Supplementary file 5 [file Table5.docx]

| Oral Species | Rho | *p*-value | 95% CI | adj *p* |
| --- | --- | --- | --- | --- |
|  |  |  | **Lower, Upper** |  |
| *Actinomyces sp. oral taxon 171* | -0.261 | <.001 | -0.41, -0.11 | .04 |
| *Aggregatibacter segnis* | -0.212 | .01 | -0.36, -0.06 | .09 |
| *Anaeroglobus geminatus* | 0.244 | .002 | 0.09, 0.39 | .06 |
| *Bifidobacterium dentium* | 0.289 | <.001 | 0.14, 0.43 | .02 |
| *Dialister pneumosintes* | 0.319 | <.001 | 0.17, 0.46 | .005 |
| *Fusobacterium nucleatum* | 0.223 | .01 | 0.07, 0.37 | .08 |
| *Haemophilus parahaemolyticus* | -0.213 | .01 | -0.37, -0.06 | .09 |
| *Haemophilus paraphrohaemolyticus* | -0.218 | .01 | -0.37, -0.07 | .08 |
| *Haemophilus pittmaniae* | -0.238 | .003 | -0.39, -0.09 | .05 |
| *Lancefieldella parvula* | 0.217 | .01 | 0.06, 0.37 | .08 |
| *Mycoplasma salivarium* | 0.234 | .003 | 0.08, 0.38 | .06 |
| *Neisseria lactamica* | -0.241 | .002 | -0.39, -0.09 | .06 |
| *Neisseria meningitidis* | -0.223 | .01 | -0.37, -0.07 | .08 |
| *Neisseria mucosa* | -0.239 | .003 | -0.39, -0.09 | .06 |
| *Neisseria polysaccharea* | -0.265 | <.001 | -0.41, -0.11 | .04 |
| *Neisseria subflava* | -0.245 | .002 | -0.39, -0.09 | .06 |
| *Parvimonas micra* | 0.325 | <.001 | 0.18, 0.47 | .01 |
| *Prevotella denticola* | 0.291 | <.001 | 0.14, 0.44 | .02 |
| *Prevotella enoeca* | 0.245 | .00 | 0.09, 0.39 | .07 |
| *Prevotella oralis* | 0.223 | .01 | 0.07, 0.37 | .07 |
| *Prevotella oris* | 0.232 | .004 | 0.08, 0.38 | .06 |
| *Pseudoleptotrichia goodfellowii* | -0.237 | .003 | -0.39, -0.09 | .05 |
| *Schaalia meyeri* | 0.248 | .002 | 0.10, 0.40 | .07 |
| *Solobacterium moorei* | 0.324 | <.001 | 0.18, 0.47 | .01 |
| *Streptococcus infantis* | -0.240 | .003 | -0.39, -0.09 | .06 |
