# Supplementary material for: An association between poor oral health, oral microbiota, and pain identified in New Zealand women with central sensitisation disorders: a prospective clinical study
Source: Front Pain Res (Lausanne). 2025 Apr 9;6:1577193. doi: 10.3389/fpain.2025.1577193 (PMC12014678; doi:10.3389/fpain.2025.1577193)
Supplement: Supplementary file 6 [file Table6.docx]

Erdrich et al., 2025. Oral Health, Oral Microbiota and Pain

**Table Legends**

**Table 1.** Characteristics of the cohort (n=158)

Key: HEI = healthy eating index, a Medians presented for results of non-parametric data only

**Table 2**. Associations between bodily pain (SF36) and relative abundance of oral species, after adjusting for age, BMI and added dietary sugar

Key: a= positively correlated with SF36-BP scores, b = inversely correlated with SF36-BP scores, Adj p = adjusted p-value after Benjamini-Hochberg correction

**Table 3**. Correlational analysis between relative abundance of oral genera and body pain scores

Key: Adj p = adjusted P-value after Benjamini-Hochberg correction

**Table 4**. Differences in relative abundance of oral genera in women with and without migraine

**Table 5**. Correlations between relative abundance of oral genera and functional bowel disorder severity scores in 156 women

Key: Adj p = adjusted P-value after Benjamini-Hochberg correction

**Supplemental tables**

**Supp Table 1**. Oral health questionnaire.

**Supp Table 2**. Correlation coefficients between relative abundance of oral microbial species and oral health scores of 156 women

**Supp Table 3.** Correlation coefficients between relative abundance of oral microbial species and oral health scores of 156 women

Key: Adj p = adjusted p-value after Benjamini-Hochberg correction

**Supp Table 4.** Generalised linear model (GLM) results for association between relative abundance of oral species and oral health scores in women

Key: OH = oral health, Adj p = adjusted P-value after Benjamini-Hochberg correction

Results of correlational analysis between pain indices and oral health

**Supp Table 5.** Correlation coefficients of relative abundance of oral species with bodily pain scores as evaluated by the SF36

Key: Adj p = adjusted p-value after Benjamini-Hochberg correction
