# Supplementary material for: An association between poor oral health, oral microbiota, and pain identified in New Zealand women with central sensitisation disorders: a prospective clinical study
Source: Front Pain Res (Lausanne). 2025 Apr 9;6:1577193. doi: 10.3389/fpain.2025.1577193 (PMC12014678; doi:10.3389/fpain.2025.1577193)
Supplement: Supplementary file 7 [file Table7.docx]

Figure 1. Recruitment flow diagram

Figure 2A. Pairwise comparisons of oral health scores according to SF-36 bodily pain score, when grouped by quintile. Participants scoring zero for the SF36-BP are compared separately. Each node shows the average rank for SF36 bodily pain by quintile. *p-values after Bonferroni correction for multiple comparisons.

Figure 2B. Scatterplot demonstrating increasing scores on the widespread pain index (range: 0 - 19) correlating with lower oral health scores (range: 147 – 226), with a regression line.

Figure 3. Differences in oral health scores by migraine status

Figure 4. Migraine headache incidence (A) and migraine sub-types (B) by oral health quintiles

*Figure 5. Comparison of oral health scores by severity groupings for functional bowel disorders, demonstrating that as oral health scores decrease, severity of functional bowel disorder-related pain increase * all P-values <.001 after Bonferroni correction for multiple comparisons.*

*Figure 6. Heatmap showing correlations between relative abundance of 29 genera and oral health, pain,* *and migraine scores. Inverse correlations are blue, positive correlations are red.*

Supplementary Figures

Supplementary Figure 1. Oral composition by genera from saliva samples

Supplementary Figure 2. Relative abundance of genera significantly correlated with migraine scores in women with and without migraine. The scales of Y-axes vary as some genera are of very low relative abundance. *Veillonella is scaled log-10 due to high abundance of this genus.

Supplementary Figure 3. Oral microbiota composition by phyla from saliva samples
