# Supplementary figures and images for: An association between poor oral health, oral microbiota, and pain identified in New Zealand women with central sensitisation disorders: a prospective clinical study
Source: Front Pain Res (Lausanne). 2025 Apr 9;6:1577193. doi: 10.3389/fpain.2025.1577193 (PMC12014678; doi:10.3389/fpain.2025.1577193)

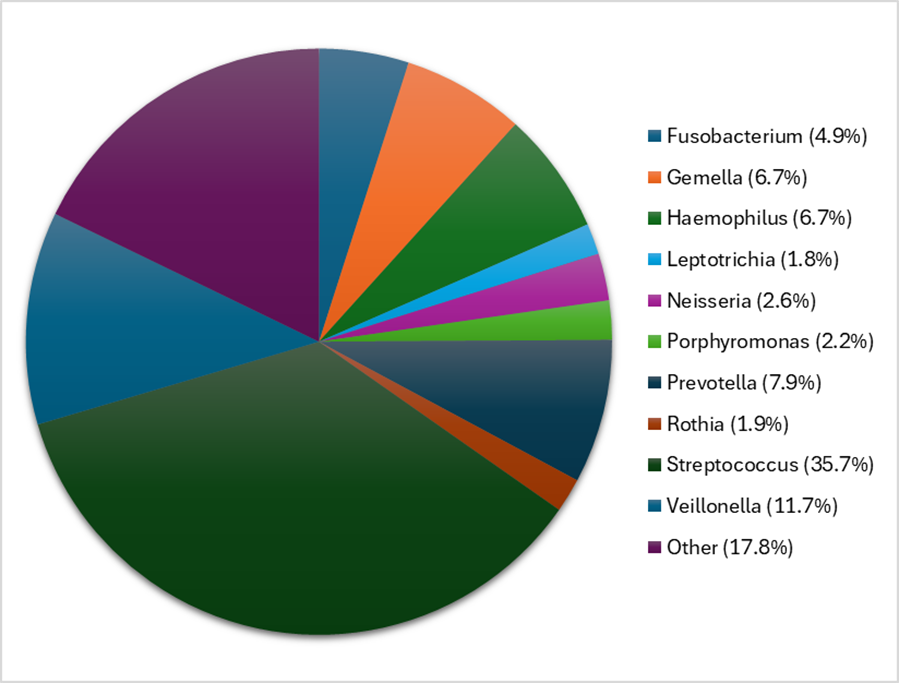

Supplement: Supplementary file 8 [file Image1.png]

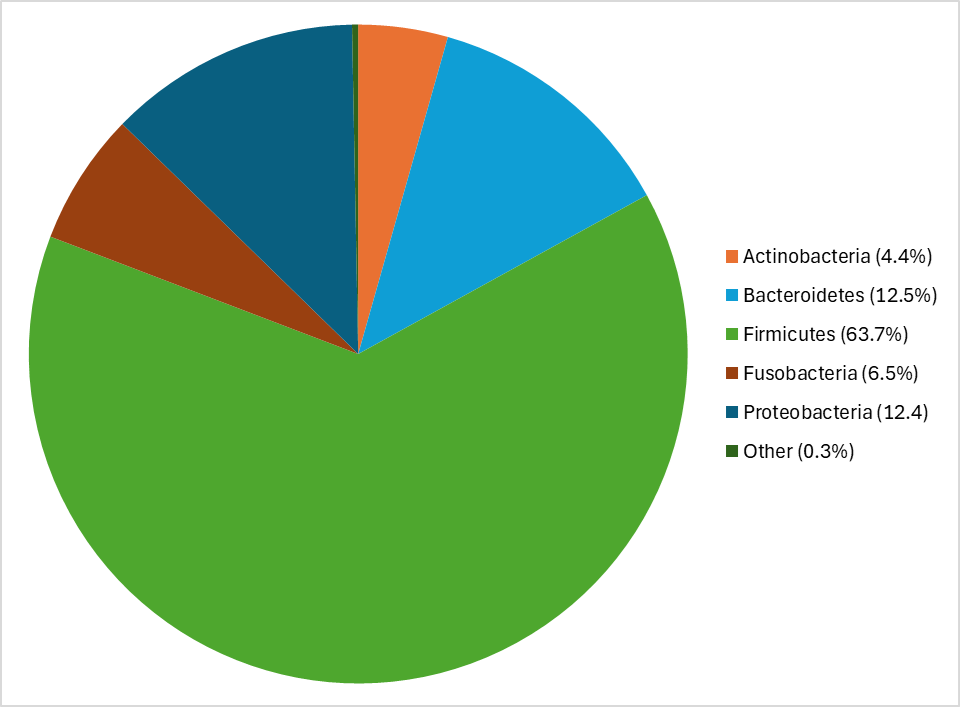

Supplement: Supplementary file 9 [file Image2.png]

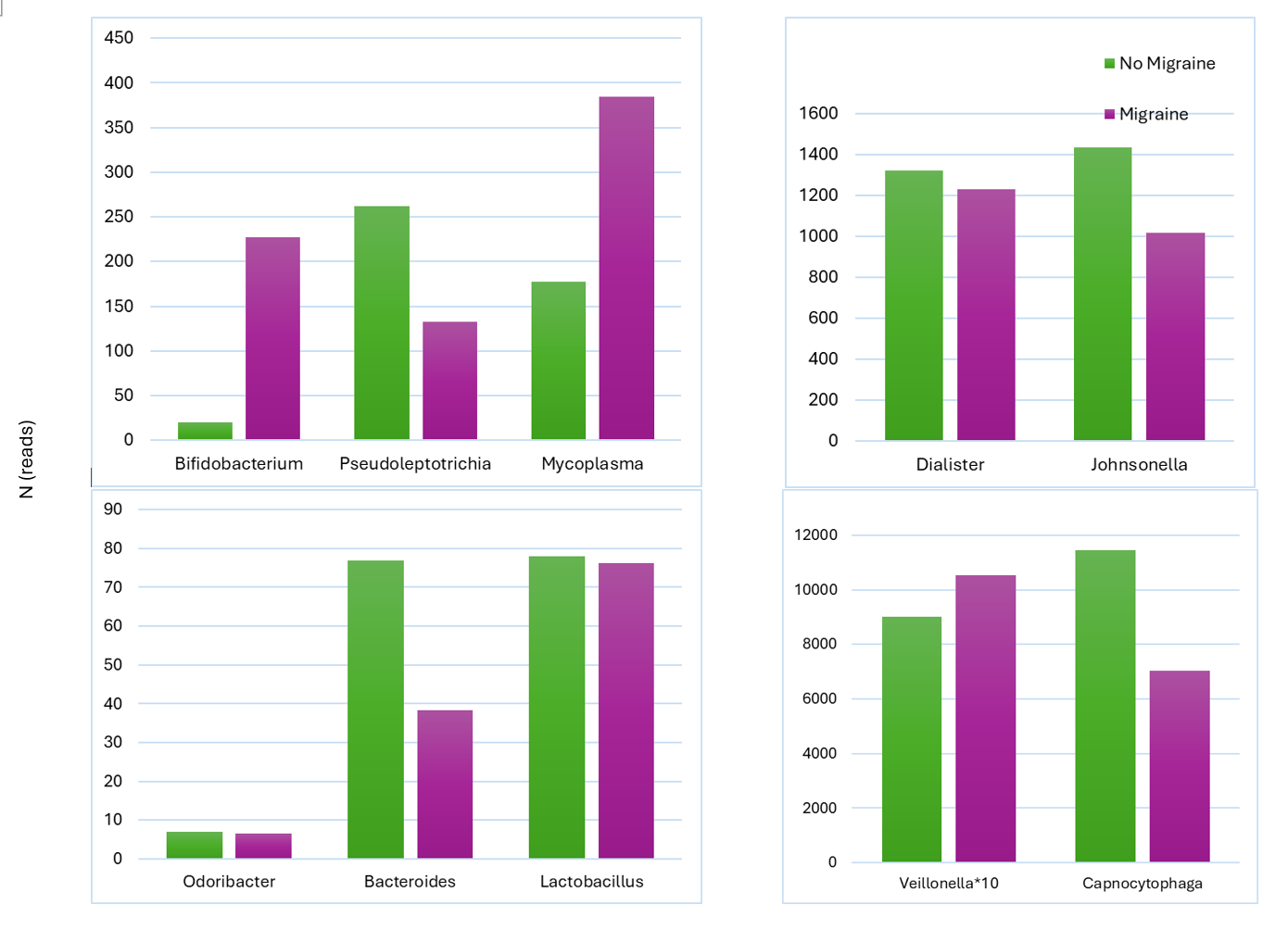

Supplement: Supplementary file 10 [file Image3.png]
